# Association of hypernatremia with mortality in patients with COVID‐19: A systematic review and meta‐analysis

**DOI:** 10.1002/iid3.1109

**Published:** 2023-12-12

**Authors:** Yongzhi Ma, Panjuan Zhang, Ming Hou

**Affiliations:** ^1^ Qinghai University Affiliated Hospital Xining China

**Keywords:** COVID‐19, hypernatremia, meta‐analysis, mortality, sodium

## Abstract

**Background:**

The COVID‐19 pandemic worldwide has caused varying degrees of severity of lung damage in patients, with acute respiratory distress and death in severe cases. However, this is not directly caused by the virus itself, but by the production of inflammasome by monocytes in the body, leading to a systemic inflammatory response, which results in a very poor clinical prognosis for patients with COVID‐19.

**Objective:**

The purpose of this meta‐analysis was to look at the relationship between hypernatremia and mortality in COVID‐19 patients.

**Methods:**

We searched the PubMed, Web of Science, Embase, and Cochrane databases for articles published from the inception of the database until August 27, 2022. Three researchers reviewed the literature, retrieved data, and assessed the quality of the literature, respectively. A meta‐analysis was performed using State 17 software to assess the value of the effect of hypernatremia on mortality in patients with new coronavirus pneumonia.

**Results:**

A total of nine publications was finally included in this study, including a total of 11,801 patients with COVID‐19, including 1278 in the hypernatremia group and 10,523 in the normonatremia group. Meta‐analysis showed that hypernatremia was associated with mortality in patients with COVID‐19 [OR = 4.15, 95% CI (2.95–5.84), *p* = .002, *I*² = 66.7%] with a sensitivity of 0.36 [0.26, 0.48] and a specificity of 0.88 [0.83, 0.91]. The posterior probability of mortality was 42% in patients with COVID‐19 hypernatremia and 15% in patients who did not have COVID‐19 hypernatremia.

**Conclusion:**

According to available data, hypernatremia is associated with death in patients with COVID‐19.

## Graphical abstract

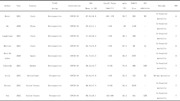

## 1 INTRODUCTION

COVID‐19 is an acute infectious respiratory disease associated with severe acute respiratory syndrome coronavirus 2 (SARS‐CoV‐2) infection. And as of September 4, 2022, the novel coronavirus has spread greatly in the population and has spread in more than 200 countries worldwide, and the World Health Organization has reported more than 600 million confirmed cases of novel coronavirus pneumonia and more than 6.4 million deaths.^1^ At present, there are several mutated strains of novel coronaviruses, and there are five major mutated strains of SARS‐CoV‐2 recognized worldwide, namely Alpha, Beta, Gamma, Delta, and Omicron,^2^ which have caused many cases, high transmission capacity, wide range, and high virulence worldwide,^3^, ^4^, ^5^, ^6^, ^7^ causing serious harm to human life and health. It is a significant burden on global health care and presents new challenges in preventing and controlling global epidemics.

Although mild to moderate symptoms are now common in patients infected with COVID‐19, critical care or respiratory support is still required for patients in a critical state. Disturbances in electrolyte levels are a common clinical phenomenon in patients suffering from COVID‐19,^8^ and severely ill patients have delicate internal environmental homeostasis and are more likely to have metabolic disorders in electrolyte levels. Hypernatremia means a plasma sodium concentration of more than 145 mmol/L.^9^ The hypertonic state caused by hypernatremia also has a tremendous impact on the organism, and the deleterious effects of this hypertonic state on patients include brain cell shrinkage, cerebral demyelination, coma, rhabdomyolysis and reduced left ventricular function^10^ and rhabdomyolysis.^11^, ^12^ The development of hypernatremia in COVID‐19 patients is usually due to fever, diarrhea, anorexia, and decreased fluid intake,^13^ in addition, studies have shown that advanced age, dementia, and institutionalization are important risk factors for the development of hypernatremia in patients with COVID‐19.^14^ Recent research indicates that the prevalence of hypernatremia in patients with COVID‐19 is between 2% and 7% and is associated with the risk of death in patients with COVID‐19,^6^, ^7^, ^8^, ^15^, ^16^ this suggests that hypernatremia is associated with mortality risk in patients with COVID‐19. Although the pathogenicity of SRAS‐CoV‐2 has decreased, there are still many patients who are still severely infected. Then early detection of patients' blood sodium levels can identify high‐risk patients who need close monitoring, so that further effective treatment options can be taken to reduce patient mortality and improve the rational allocation of healthcare resources. Therefore, in this study, we used systematic assessment and meta‐analysis to evaluate the impact of hypernatremia on mortality in patients with COVID‐19.

## 2 METHODS

This is a Preferred Reporting Items for Systematic Reviews and Meta‐Analyses (PRISMA) guidelines compliant meta‐analysis. This meta‐analysis is registered at PROSPERO (PROSPERO ID: CRD42021233592).

### 2.1 Eligibility criteria

Studies meeting the following criteria were included: (1) retrospective and prospective cohort studies, (2) patients with confirmed diagnosis of novel coronavirus pneumonia (3) hypernatremia and normonatremia (4) patients ≥18 years of age. Studies meeting the following criteria were excluded: (1) review‐type literature, (2) case reports, (3) conference abstracts, (4) duplicate reports, (5) literature with missing data, and (6) pregnant women. Pregnant women were excluded to reduce bias due to physiological status and changes in glomerular filtration and renal plasma flow that are specific to the general population.

### 2.2 Search strategy and study selection

Two researchers systematically searched PubMed, Web of Science, Embase, and Cochrane literature databases for studies of hypernatremia associated with patients with COVID‐19 through August 2022, and we used a combination of subject terms and free words to search for keywords including: “COVID‐19, 2019‐nCoV Disease, COVID‐19 Virus Infection, etc., Hypernatremia, hypernatraemia, Hypernatremias, hypernatriaemia, hypernatriemia.” The retrieved articles were managed using EndNote X9.1, and the relevant literature cited in these articles was read.

### 2.3 Data extraction

First, three researchers independently read the document titles and summaries, reviewed the required documentation against inclusion and exclusion criteria, and extracted the data for analysis. If disagreements were identified during the screening process, the issues were addressed through discussion or expert advice. Data were noted using a Microsoft Excel worksheet, including first author, country, time of publication, type of study design, sample size, age (Mean ± SD), male (%), hypernatremia cutoff range, length of stay, ICU admission, and mortality.

### 2.4 Literature quality screening and quality evaluation

Two investigators independently assessed the quality of the referenced literature using the Newcastle‐Ottawa scale (NOS), and consulted a third party to solve the problem when there was bias in the results. NOS consisted of three main aspects: study population selection, comparability between groups, and exposure evaluation or outcome evaluation. The score is 9 out of 9. Studies of low quality: 0–3, studies of average quality: 4–6, and studies of high quality: 7–9.

### 2.5 Statistical analysis

We conducted a statistical analysis of the included literature using the State 17 software. Effect value indicators for the effect of hypernatremia on mortality in patients with novel coronavirus pneumonia were expressed using the ratio (OR), 95% confidence interval (95% CI), with *p* < .05 indicating a statistically significant difference. For the test of heterogeneity *I* ^2^ > 50%, *p* ≤ .1, suggesting significant heterogeneity, Meta‐analysis was performed using the random‐effects model, considering the source of their heterogeneity and dealing with it; for the test of heterogeneity *I* ^2^ ≤ 50%, *p* > .1, indicating relatively little heterogeneity among studies, the fixed‐effects model was used. The included documentation was analyzed for release bias using the State 17 software Egger test.

## 3 RESULTS

**Figure 1:**
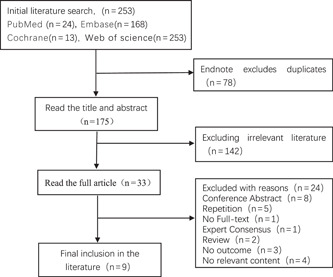
Screening flowchart.

Initially, 253 publications were researched, including 24 in PubMed, 48 in Web of Science, 168 in Embase, and 13 in Cochrane, with articles published up to August 27, 2022. Seventy‐eight duplicates were removed using Endnote software, then the titles and abstracts of the literature were read according to the inclusion and exclusion criteria, 142 irrelevant papers were excluded, and finally, the contents of the remaining 33 papers were carefully read, and a total of 9 matching papers were included^17^, ^18^, ^19^, ^20^, ^21^, ^22^, ^23^, ^24^, ^25^ (Figure 1). The nine included studies, seven of which were retrospective cohort studies^17^, ^18^, ^20^, ^21^, ^22^, ^23^, ^25^ and two prospective cohort studies,^19^, ^24^ included a total of 11,801 patients suffering from COVID‐19, including 10,523 patients with normal blood sodium and 1278 patients with hypernatremia, with an incidence of 10.8% hypernatremia and a mortality rate of 48.7%. Among these studies, five studies^19^, ^20^, ^23^, ^24^, ^25^ reported the relationship between hypernatremia and ICU admission, including a total of 1348 patients with COVID‐19, of whom 444 were admitted to the ICU. As well, seven studies^18^, ^19^, ^20^, ^21^, ^23^, ^24^, ^25^ reported the relationship between hypernatremia and duration of stay. A study documented 30‐day mortality among hospitalized patients.^19^ The included literature was scored according to the NOS scale, with one literature scoring 5, two scoring 6, five scoring 7, and one scoring 8. The overall quality of the literature was good. The fundamental features of the included studies are illustrated in Table 1.

**Table 1:** Basic characteristics of the literature and quality scores.

Author	Year	Country	Study design	Intervention	Age (Mean ± SD)	Cutoff value (mmol/L)	Male (%)	Sample size	ICU admission	Outcome	NOS
Berni	2021	Italy	Retrospective	COVID‐19	67.0 ± 16.8	146–170	59.7	293	60	In‐hospital mortality	6
Hu	2020	China	Retrospective	COVID‐19	55.2 ± 11.1	>145	50	1130	–	In‐hospital mortality	7
Longhitano	2021	Italy	Retrospective	COVID‐19	71.0 ± 16.4	>145	48.1	106	–	In‐hospital mortality	7
Martino	2021	Italy	Retrospective	COVID‐19	63.7 ± 13.0	>145	67.4	86	11	In‐hospital mortality	6
Ruiz‐Sánchez	2020	Spain	Retrospective	COVID‐19	61.7 ± 21.5	>145	56.5	3707	–	In‐hospital mortality	8
Sjöström	2021	Sweden	Retrospective	COVID‐19	58.2 ± 13.7	≥145	74.9	406	146	In‐hospital mortality	7
Atila	2021	Switzerland	Perspective	COVID‐19	53.6 ± 17.9	>145	52.5	122	22	30‐day mortality	5
Hirsch	2021	United States	Retrospective	COVID‐19	66.9 ± 17.3	≥145	54.4	5510	–	In‐hospital mortality	7
Yen	2022	United States	Perspective	COVID‐19	60.3 ± 18.1	142–169	44.2	441	128	In‐hospital mortality	7

The definition and extent of hypernatremia were measured in seven of the nine included studies that measured blood sodium levels on admission, and one study recorded peak blood sodium levels during hospitalization in patients with COVID‐19.^25^ One study recorded the blood sodium levels of hospitalized patients using a standard sample form.^17^

### 3.1 Meta‐analysis results

**Figure 2:**
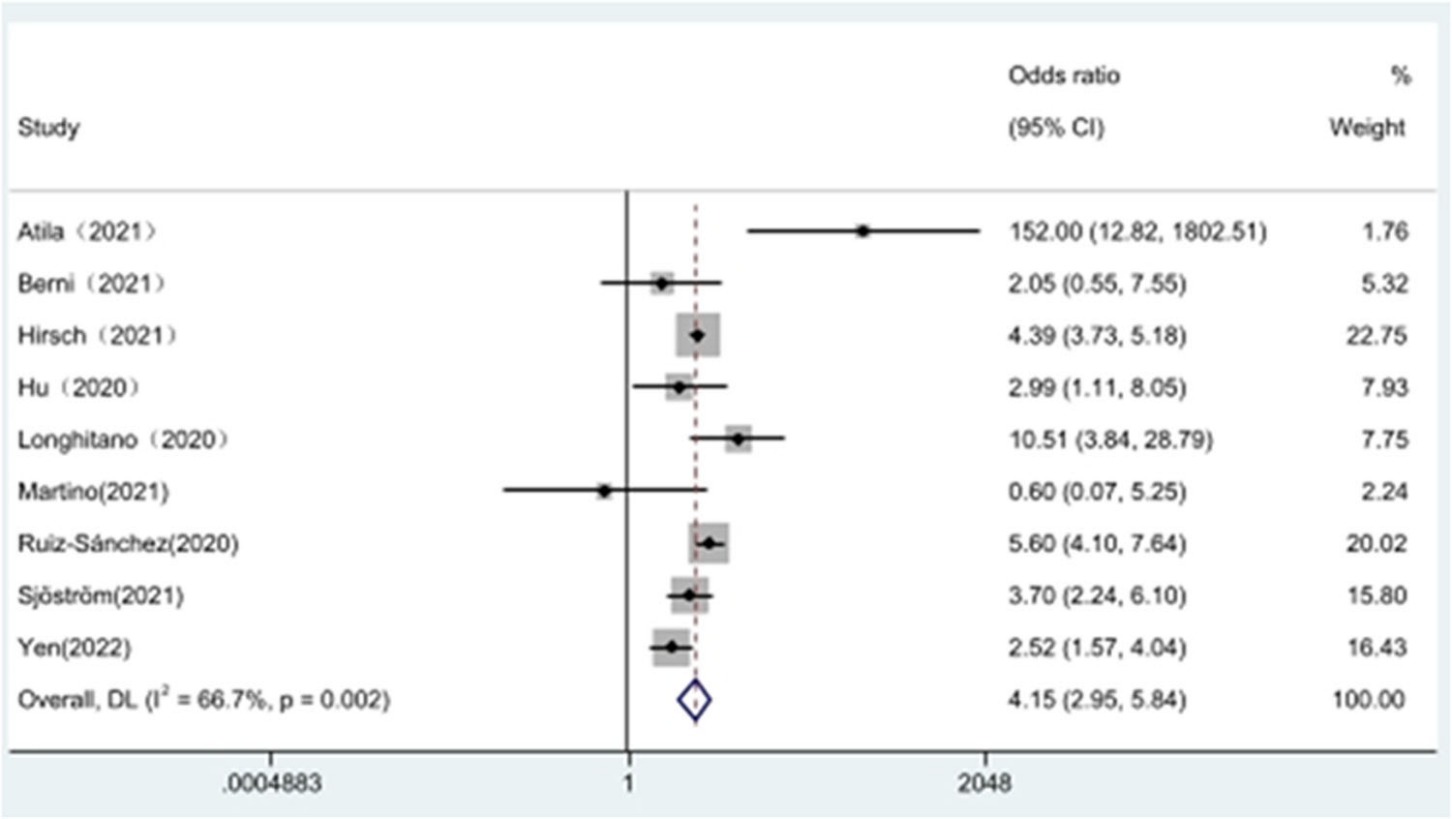
Hypernatremia and mortality.

**Figure 3:**
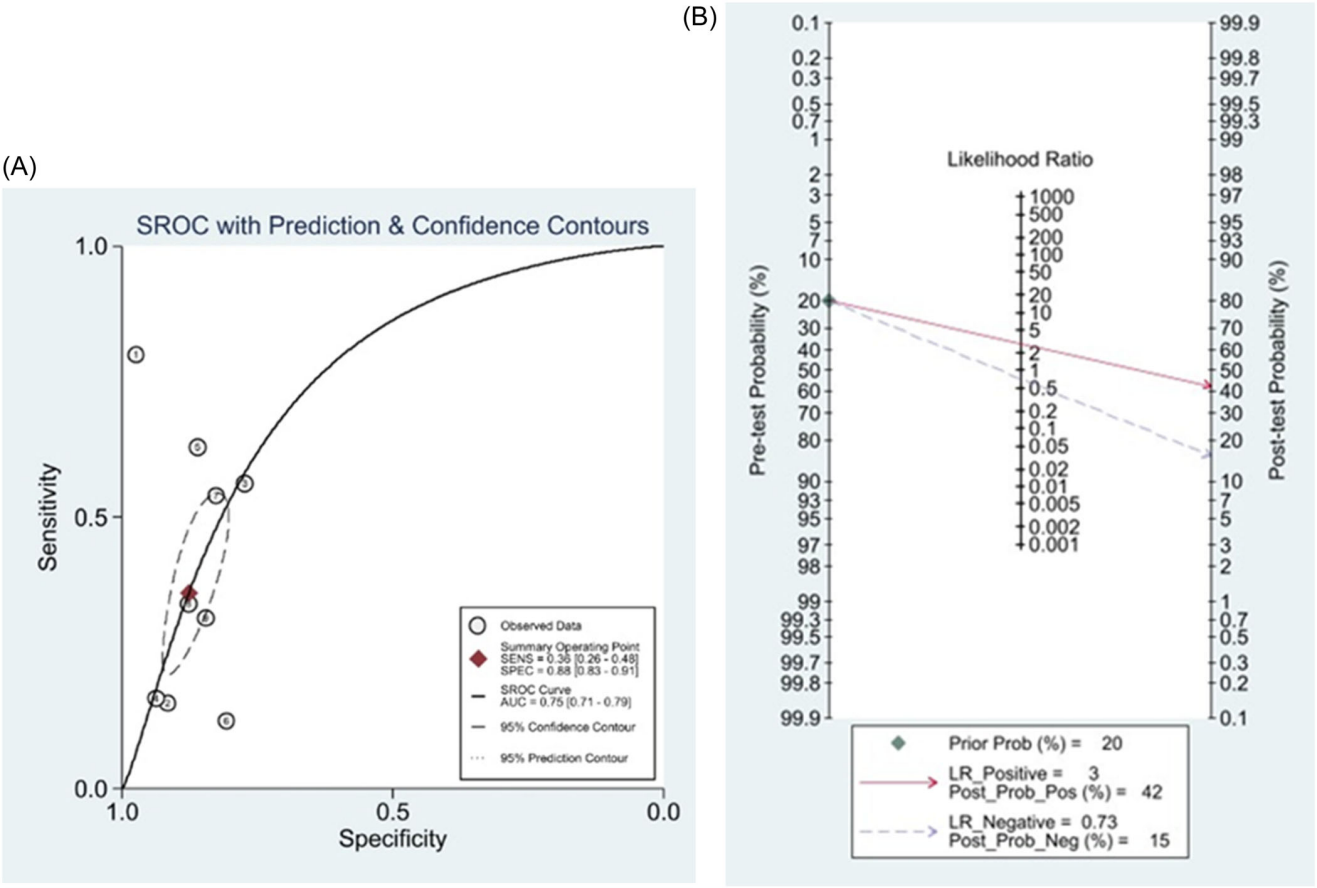
(A) SROC curve. (B) Fagan's nomogram.

Analysis of the association between hypernatremia and mortality in patients with novel coronavirus pneumonia using a fixed effects model [OR = 4.31, 95% CI (3.78–4.90), *p* = .002, *I*² = 66.7%] showed significant heterogeneity between the included studies by heterogeneity test. Consequently, the included studies were reanalyzed using a randomized model, and the results showed that hypernatremia was associated with mortality in COVID‐19 patients [OR = 4.15, 95% CI (2.95–5.84), *p* = .002, *I*² = 66.7%], With a significant statistical difference, as illustrated in Figure 2. The SROC curve generated according to hypernatremia showed a sensitivity of 0.36 [0.26, 0.48], specificity 0.88 [0.83, 0.91], and AUC 0.75 [0.71, 0.79], see Figure 3A. The Fagan curve showed a posterior probability of 42% mortality in COVID‐19 hypernatremia patients and 15% mortality in non‐COVID‐19 hypernatremia patients, see Figure 3B. Additionally, we performed a sensitivity analysis of the included documentation using the STATE 17 software, and the results showed that the meta‐analysis results were stable. We also used the Egger test STATE 17 software to detect publication bias in the literature, and the test result: P > |t| indicator was 0.92, and there was no publication bias in this included study.

### 3.2 Heterogeneity test

**Figure 4:**
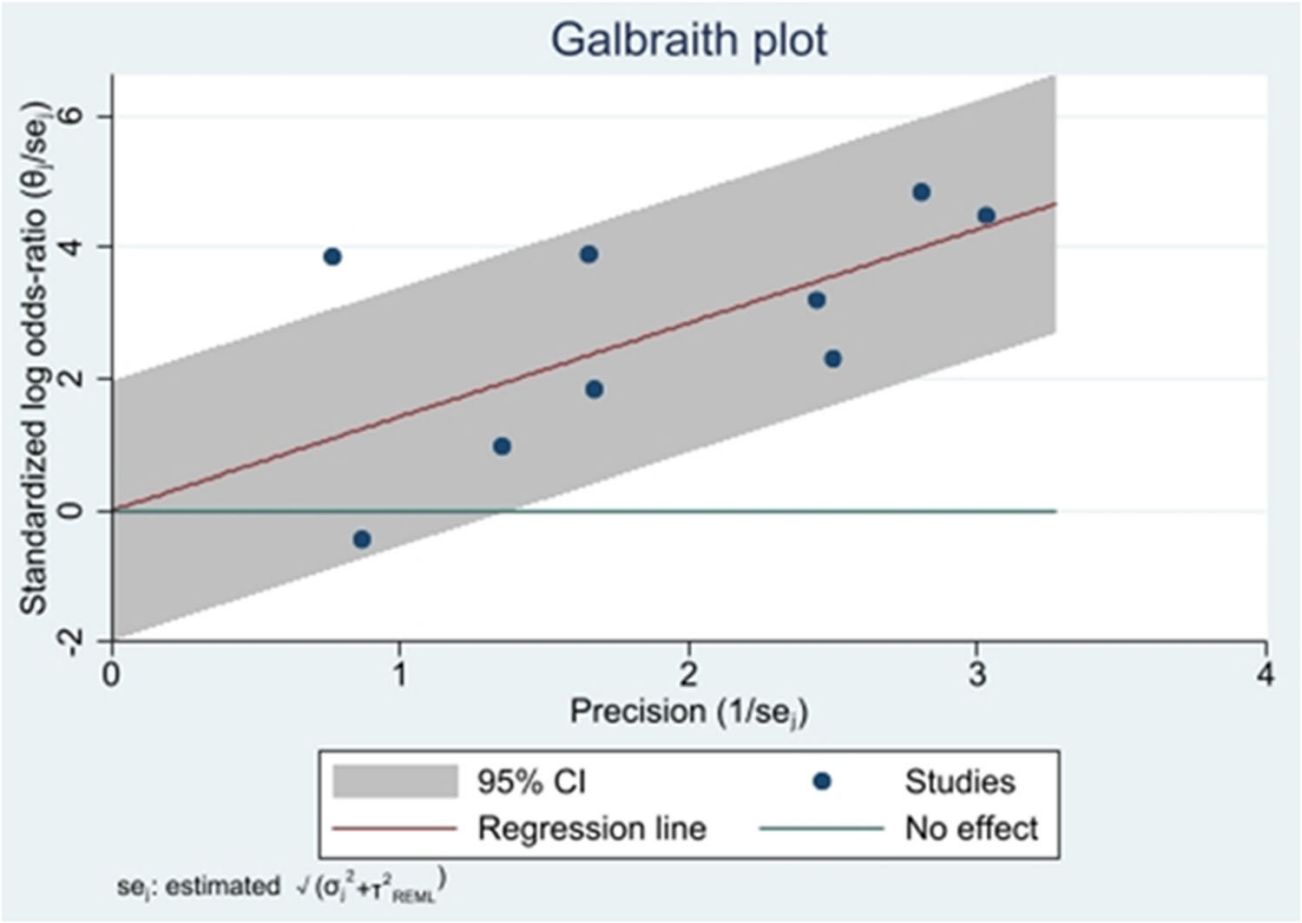
Galbraith plot.

To explore the source of heterogeneity, we have plotted Galbraith using Stata 17 software, see Figure 4. It can be clearly observed that one of the original studies^19^ in the Galbraith plot fell into the 95% CI regression line outside, with a strong heterogeneity between it and other studies, and all other points fall in the 95% CI regression line. The heterogeneity between the studies decreased by 10.2% with the exception of the Atila study,^19^ suggesting that this study may be one of the main sources of heterogeneity. Furthermore, we found an equally significant decline in the heterogeneity (down 4.7%) after excluding the yen study.^24^

### 3.3 Subgroup analysis

**Figure 5:**
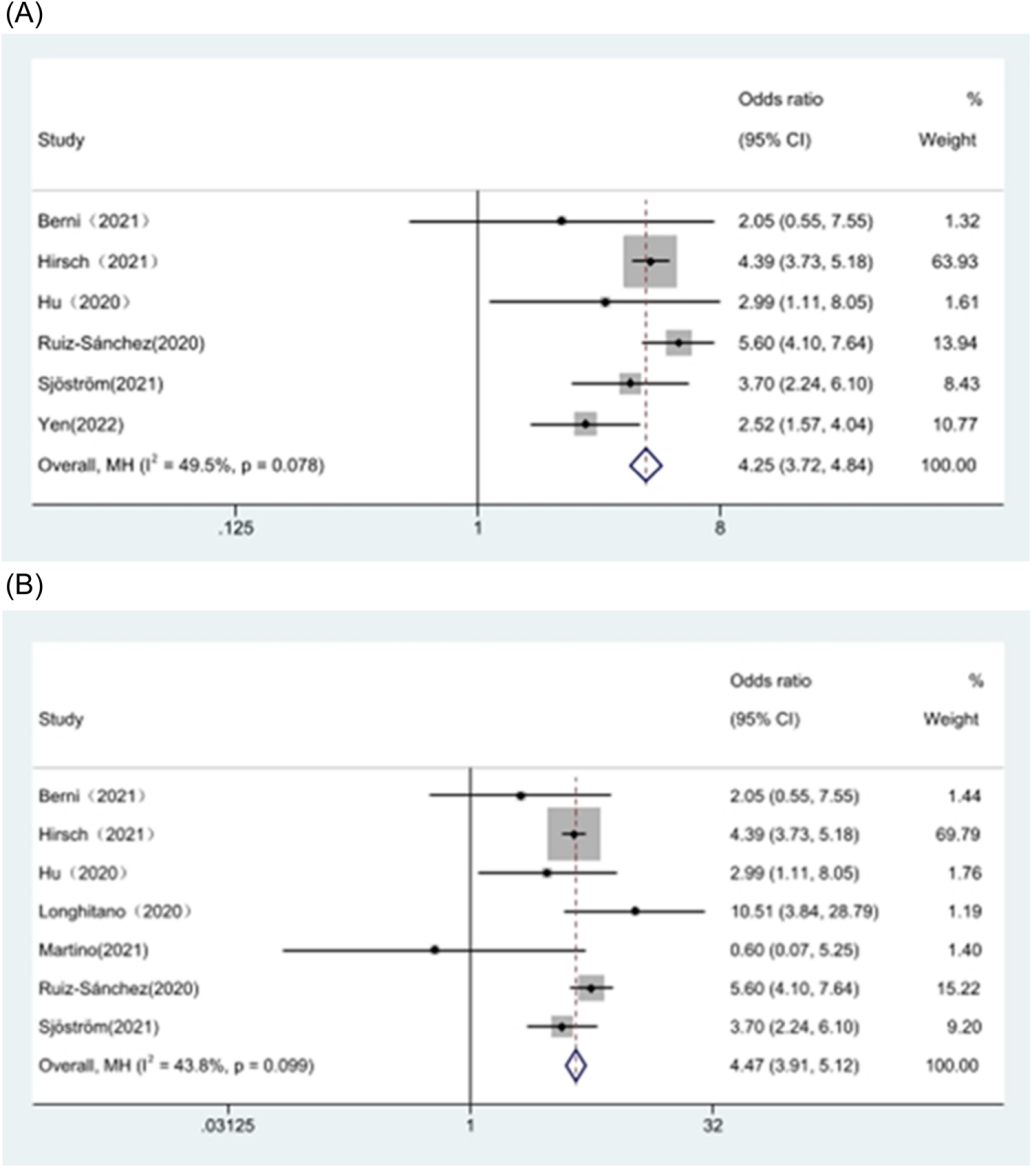
Subgroup analysis (A) Sample size >200 (B) Retrospective study.

Given the large study heterogeneity (*I*² = 66.6%), we performed separate subgroup analyses for sample size (>200 cases) and study type (retrospective cohort study), which showed a significant decrease in heterogeneity. Analysis of subgroups of studies that have a sample size greater than 200 cases is presented in Figure 5A. The results showed that hypernatremia was associated with mortality in patients with COVID‐19 (OR = 4.25, 95% CI 3.72–4.84, *p* < .05). We then analyzed subgroups from seven retrospective cohort studies, see Figure 5B, and again, there was a significant association (OR = 4.47, 95% CI 3.91–5.12, *p* < 0.05). We did not perform subgroup analyses prospective studies alone, as these two original papers were the main source of heterogeneity.^19^, ^24^

**Figure 6:**
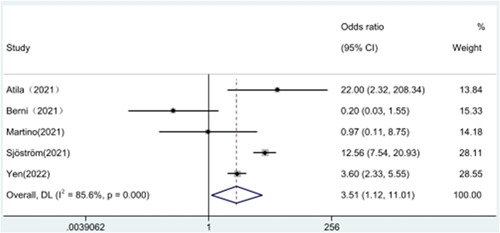
Hypernatremia and ICU admission.

We also analyzed the relationship between hypernatremia and admission to the ICU. A total of five original papers^19^, ^20^, ^23^, ^24^, ^25^ documented ICU admissions in patients with COVID‐19, and due to the large heterogeneity of the analysis using a fixed‐effects model (*I*² = 85.7%), a random‐effects model analysis was used, which showed an association between hypernatremia and ICU admission (OR = 3.51, 95% CI 1.12–11.01, *I*² = 85.6%), (Figure 6).

## 4 DISCUSSION

In this systematic assessment and meta‐analysis, a total of 11,801 COVID‐19 patients was included to assess the effect of hypernatremia on mortality in COVID‐19 patients. The included studies came from six countries, including China, the United States, Italy, Switzerland, Sweden, and Spain, and the vast majority of COVID‐19 patients came from the United States. Our statistical analysis of the studies showed that the proportion of deaths in the normonatremia group of patients with COVID‐19 (1898/10,523) was much lower than the proportion of deaths in the hypernatremia group of patients with COVID‐19 (622/1278). The occurrence of hypernatremia was associated with mortality in COVID‐19 patients compared with COVID‐19 patients who did not develop blood sodium abnormalities during hospitalization [OR = 4.31, 95% CI (3.78–4.90), *p* = .002, *I*² = 66.7%], with a sensitivity of 0.36 [0.26, 0.48], specificity of 0.88 [0.83, 0.91], with an AUC of 0.75. Moreover, our study also showed a significant effect of the development of hypernatremia on admission to the ICU.

COVID‐19 pandemics around the world are primarily caused by SARS‐CoV‐2 infections, and the clinical presentation can range from asymptomatic to mild upper respiratory tract infections or severe lung injury. SARS‐CoV‐2 infection induces a cytokine storm (CS) leading to the release of proinflammatory cytokines, such as interleukin (IL)−6, IL‐1β, and tumor necrosis factor (TNF)‐α, which play an important role in the progression of COVID‐19, and the mechanism is mainly related to the interaction between SARS‐CoV‐2 and immune cells.^26^, ^27^ According to research, COVID‐19 severity and clinical prognosis are strongly linked to CS.^28^

Studies have shown that SARS‐CoV‐2 can cause damage to the respiratory, neurological, cardiovascular, gastrointestinal, and genitourinary systems of patients, and its most common clinical symptoms include fever, cough, malaise, anorexia, and shortness of breath.^29^ There are multiple pathophysiological mechanisms that lead to Hypernatremia in patients with COVID‐19, whose gastrointestinal tract and kidneys play a critical role in disrupting the water‐electrolyte balance.^30^ Previous studies have shown that SARS‐CoV‐2 enters the body mainly through binding to the host angiotensin‐converting enzyme 2 (ACE2) receptor,^31^ and a large number of ACE2 receptors are present in renal cells, with nearly 100‐fold more expression in the kidney and gastrointestinal tract than in the lungs,^32^ leading to renal tissue damage in COVID‐19 patients, and the kidney is involved in the regulation of water‐electrolyte balance regulation, and its impairment causes fluid and electrolyte imbalance.^33^ Many studies have also demonstrated that the onset of kidney failure in patients with COVID‐19 is relatively frequent.^34^, ^35^, ^36^

Second, COVID‐19 patients are predisposed to gastrointestinal problems, with major symptoms including diarrhea, nausea, vomiting, and abdominal pain.^37^ A bibliometric analysis showed that 2.6%–75% of COVID‐19 patients develop gastrointestinal symptoms during infection,^38^ and the mechanism causing gastrointestinal disorders can be explained by the pathophysiology that SARS‐CoV‐2 can bind to ACE2 receptors on intestinal cells,^39^ which are highly expressed in proximal and distal enterocytes, disrupting the normal intestinal mucosa and causing diarrhea, absorption, and other dysfunctions. In addition, SARS‐CoV‐2 can also disrupt the intestinal flora by binding to ACE2 receptors, leading to diarrhea. These conditions may result in increased water loss from the machine, which may result in increased blood sodium.

Insensible water loss due to fever and shortness of breath can cause the development of hypernatremia in patients. The respiratory tract and skin are the main sources of insensible water loss, a normal adult loses approximately 1000 mL of water per day through the respiratory tract and skin, and in the case of fever, fluid loss through the skin will increase by 3‐5 mL/kg/day for every 1°C rise in body temperature. The most common symptom in patients with COVID‐19 is fever, which is prevalent in up to 90% of cases,^40^ and may be accompanied by diarrhea, anorexia, and tachypnea that increase fluid loss. Two studies from the Netherlands and Belgium showed that patients in the COVID‐19 hypernatremia group all had a mean urine osmolality greater than 600 mOsm/L and a normal blood volume,^13^, ^41^ which may be a very plausible explanation for the fact that hypernatremia is caused by increased fluid loss. In addition, COVID‐19 infection has the greatest impact on the lungs and can result in pulmonary edema, which may be due to impaired breakdown of bradykinin and its metabolites.^42^ To avoid exacerbation of pulmonary edema, clinicians will limit fluid administration, which could also lead to hypernatremia.

Studies have shown an association between the development of hypernatremia in COVID‐19 patients and admission to the ICU. A large number of previous studies have shown that hypernatremia is a common clinical phenomenon in the ICU,^43^, ^44^ so it is not surprising that the development of hypernatremia in patients with COVID‐19 is associated with ICU admission. A recent study suggests that hypernatremia is a predictor of death in COVID‐19 patients, that the occurrence of hypernatremia at any time during a patient's hospitalization is associated with an increased risk of death, and similarly suggests that hypernatremia is associated with LOS and the need for intensive care.^45^

Of the nine original papers we included, because the definition of patient mortality in Atila's literature was different from the rest of the literature, which may have influenced the results of the study,^19^ however, we did not include this one paper when we did the subgroup analysis and the conclusions drawn were not altered. In addition, we found that the findings of the study by Martino differed from the rest of the literature,^23^ where the mortality rate for the development of hypernatremia was lower than that of the normal blood sodium group, probably because of the small total sample size (total sample size = 86) and the insufficient sample size of COVID‐19 patients who developed hypernatremia. Since the study by Atila^19^ was the main source of heterogeneity, our analysis of this literature revealed that the small sample size (sample size = 5) and the high positivity rate (4/5) of COVID‐19 patients with hypernatremia may have contributed to the large heterogeneity.

This study also has some limitations; first, although our findings showed an association between hypernatremia and mortality in patients with COVID‐19, this does not indicate a causal relationship. Second, this study mainly involved retrospective clinical studies, and we were unable to analyze the severity of the disease in admitted patients and postadmission treatment measures, so there may be heterogeneous in the severity of disease and treatment options, which may affect the results of meta‐analysis.

In conclusion, the available data suggest a relationship between hypernatremia and mortality in patients with novel coronavirus pneumonia and an impact on ICU admission in patients with COVID‐19. In the future, it is important for physicians to identify risk factors for hypernatremia at an early stage in clinical practice, and patients who are already hypernatremia at the time of admission should be monitored and treated with a view to reducing hypernatremia in patients and decreasing mortality in patients with COVID‐19.

## AUTHOR CONTRIBUTIONS

**Yongzhi Ma**: Study design; data collection; data analysis; writing. **Panjuan Zhang**: Data collection; data analysis; writing. **Ming Hou**: Study design.

## CONFLICT OF INTEREST STATEMENT

The authors declare no conflict of interest.

## Data Availability

Indicate where the review protocol can be accessed: https://www.crd.york.ac.uk/PROSPERO/
